# Histoplasma Pyomyositis in a Patient with Disseminated Histoplasmosis and Anti-Synthetase Syndrome: Case-Based Review of Literature

**DOI:** 10.31138/mjr.31.3.350

**Published:** 2020-09-30

**Authors:** Harikrishnan Gangadharan Nair, Amita Aggarwal, R Naveen, Rungmei SK Marak, Rachna Agarwal, Latika Gupta

**Affiliations:** 1 Departments of Clinical Immunology and Rheumatology; 2 Microbiology and; 3 Ophthalmology Sanjay Gandhi Post Graduate Institute of Medical Sciences, Lucknow, India

**Keywords:** Histoplasma capsulatum, myositis, pyomyositis, anti-synthetase syndrome, histoplasmosis

## Abstract

**Background:**

Histoplasmosis is an endemic mycosis caused by Histoplasma Capsulatum, a thermally dimorphic fungus with mycelial and yeast forms. Muscle involvement is infrequent in Histoplasmosis.

**Case:**

A 49-year-old lady presented with generalized myalgia and arthritis of two-year duration, which had responded partly to glucocorticoids. The lady reported to us two years into the illness with ulcerative eyelid lesions, worsening myalgia, and painful skin nodules. Eventually, it turned out that anti-synthetase syndrome was the primary diagnosis with Histoplasma infection in the muscles, subcutaneous tissue, and eye. We herewith present the course of her illness and a review of Histoplasmosis of the muscle in literature.

**Conclusion:**

The differential diagnosis of painful muscle weakness is broad. Histoplasma capsulatum infection should be considered in immunosuppressed myositis patient presenting with orbital ulcers, skin nodules and worsening muscle weakness.

## INTRODUCTION

**Figure 1.**
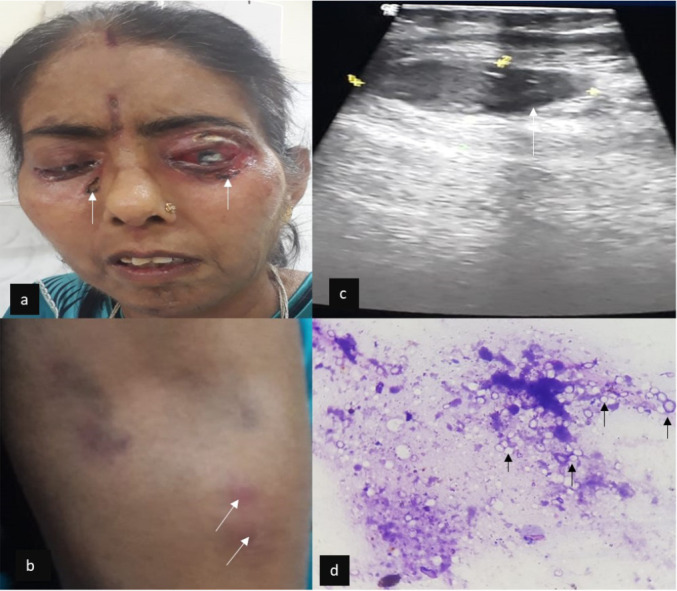
(a) Punched out ulcers on bilateral lower eyelids (white arrows), corneal ulceration and chemosis of left eye; (b) Erythematous skin nodules on left forearm (white arrows); (c) Ultrasound of left arm showing 4.88 × 1 cm hypoechoic lesion (white arrow) with loss of fibrillar architecture of muscle; (d) Giemsa stained smear of pus from eye ulcer showing numerous budding yeast cells suggestive of Histoplasma (black arrows).

A 49-year-old lady presented with generalized myalgias and arthritis of two-year duration, which had responded partly to glucocorticoids. She reported to us in December 2018, two years into the illness; with worsening myalgia and ulcerative eyelid lesions following a glucocorticoid taper. Eventually, it turned out that anti-synthetase syndrome was the primary diagnosis with Histoplasma infections in the muscles and eye (**Figure 1**). We herewith present the course of her illness and a review of Histoplasmosis of the muscle in literature.

## CASE

A 49-year-old lady presented with subacute onset, gradually progressive painful weakness of proximal muscles of bilateral upper and lower limbs for two years. During the same period, she also developed inflammatory polyarthritis of small joints. Later the same year, she developed progressive dyspnoea and biventricular systolic dysfunction (ejection fraction 34%). Dilated cardiomyopathy could explain the cardiac symptoms. However, she continued to have pedal oedema and transaminitis despite diuretics, leading to a suspicion of chronic liver disease. Anti-smooth muscle antibody came positive; and with a diagnosis of autoimmune hepatitis, 50 mg oral prednisolone was initiated 16 months after the onset of the first symptom.

Myalgia and arthritis improved with glucocorticoids over the next 8 months. However, 6 months after glucocorticoid initiation (2 months before her presentation to our hospital), she developed a painful ulcer on the left lower eyelid, which gradually extended to the corneal surface with resultant vision loss. Steroid-induced diabetes was likely, given an Hba1c of 6.5%. A fortnight later, a similar ulcer developed on the right lower eyelid though without corneal involvement. Fifteen days before presenting to us, she noticed numerous painful nodular lesions on arms, thighs and upper back, alongside worsening myalgia and as well as proximal muscle weakness.

At the time of presentation, she did not report a history of oral ulcers, rashes, photosensitivity, alopecia, Raynaud’s phenomenon, dry cough or intake of drugs known to cause muscle weakness, thyroid dysfunction, weight loss, dysphagia, dysphonia, or bleeding manifestations. Upon examination, she had a punched-out ulcer which exuded purulent secretions on left lower eyelid margin, with lid oedema and corneal opacity. A punched-out ulcer was present below the right eyelid. Lagophthalmos was present on the left, and only the projection of light was present. Vision was preserved on the right side. Numerous (n=6) tender erythematous nodules were present on both forearms, arms, thighs and upper back. Proximal muscles of all limbs were exquisitely tender to touch, excluding the feasibility of a formal muscle power examination. Examination of joints and other systems was largely non-contributory.

At this point, the differential diagnosis was broad-ranging from lupus and inflammatory myositis to undifferentiated connective tissue disease, accounting for the two-year illness manifesting as arthritis, muscle weakness and ANA positivity. However, given the disabling muscle pain and nodules, and ulcerative eye lesions, an infective pathology could not be ruled out.

Investigations (**Table 1**) revealed mild anaemia with elevated transaminases (Aspartate aminotransferase>Alanine aminotransferase) and normal alkaline phosphatase (ALP). Lactate dehydrogenase (LDH) was elevated, although serum creatinine kinase (CK) was normal. Anti-nuclear antibodies (ANA) was 2+ coarse speckled and cytoplasmic by indirect Immunofluorescence (IIF). Anti-dsDNA antibodies were negative, and complements were normal. A smear of the pus from the left eyelid ulcer revealed pus cells without bacterial or fungal elements.

**Table 1.** Laboratory Data.

**Variable**	**Reference Range**	**Day of admission**

Haemoglobin, g/dl	12–14	10.7
White blood cell count, per mm^3^	4–10	11400
**Differential count, %**		
Polymorphonuclear cells	55–70	79
Lymphocyte	20–40	15
Monocyte	2–8	2
Eosinophils	1–4	4
Platelet count, L per mm^3^	1.5–4	2.99
Aspartate aminotransferase, U/L	5–40	144
Alanine aminotransferase, U/L	5–40	103
Alkaline phosphatase, U/L	40–150	115
Creatinine(mg/dl)	0.6–1.2	1.1
Creatinine kinase, U/L	25–192	20
Lactate Dehydrogenase, U/L	85–450	619
ANA, IF		2+ coarse speckled and cytoplasmic
		<10
Anti-ds DNA, IU/L	<10	Anti Ro 52+, anti- Jo-1
ENA		40
ESR, mm/first hour	0–20	

Aspiration of nodular lesions on the forearm yielded pus, Giemsa stain of pus showed numerous budding yeast cells suggestive of Histoplasma Capsulatum. The skin biopsy from the lower eyelid ulcer yielded granulomatous inflammation. Giemsa stain again showed numerous budding yeast cells suggestive of Histoplasmosis (**Figure 1**). Ultrasound of both thighs and arms at the sites of tenderness suggested multiple hypoechoic lesions with loss of standard fibrillar architecture of muscles (**Figure 1**). Thus, the patient had pyomyositis, and sonographic drainage confirmed the presence of pus. A Giemsa stained smear showed numerous budding yeast cells suggestive of Histoplasma capsulatum.

Thus, the patient had disseminated Histoplasmosis. The patient received intravenous liposomal Amphotericin B (0.75 mg/kg) for two weeks, and oral Itraconazole (200 mg BD). However, despite the best efforts, her vision in the left eye could not be salvaged, as the cornea was involved with the formation of a false cornea and exuberant pus in the anterior chamber.

After antifungal therapy, muscle tenderness and nodular skin lesions resolved over eight weeks. The differential diagnosis for the underlying connective tissue disease included, idiopathic inflammatory myopathy (IIM), specifically anti-synthetase syndrome with significant muscle involvement, overlap myositis, and systemic lupus erythematosus (SLE). She had normal dsDNA and complements, and ANA was 2+ cytoplasmic with coarse specked nuclear pattern. She did not have any other features to suggest a diagnosis of SLE. Underlying autoimmune hepatitis was suspected due to the presence of anti-smooth muscle antibody (SMA). However, muscle disease seemed more likely with a normal echogenicity of the liver (on ultrasound) and the observed pattern of transaminitis (SGOT>SGPT) in this patient.

After being discharged from the hospital at the six-month follow-up visit, despite antifungal therapy, she had significant proximal muscle weakness, raised muscle enzymes, and manual muscle testing (MMT) score of 61 out of a total of 80. The myositis antibodies and extractable nuclear antigens (ENA) were tested at this juncture (by Line immunoblot), and turned out to be positive for anti-Jo-1 and anti-Ro-52. Muscle biopsy suggested mild perifascial lymphomononuclear inflammatory infiltrates, and fungal elements were negative. Thus, the diagnosis was revised to IIM, specifically anti-synthetase syndrome with significant muscle involvement. High-resolution CT of the chest was normal, excluding interstitial lung disease (ILD). The dilated cardiomyopathy in this patient could be attributed to Histoplasma myocarditis or cardiac involvement related to the anti-synthetase syndrome.^1,2^ A cardiac MRI showed dilated ventricles (left>right) suggesting dilated cardiomyopathy, without (abnormal) late gadolinium enhancement. However, the study was suboptimal due to poor breath-holding and irregular heart rate precluding a definite conclusion.

**Figure 2.**
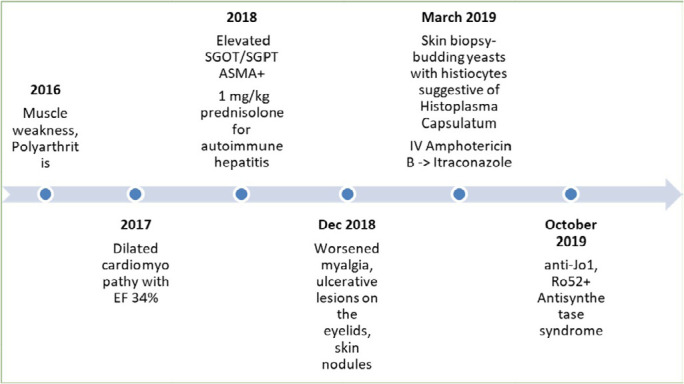
Course of illness.

## METHODS

**Figure 3.**
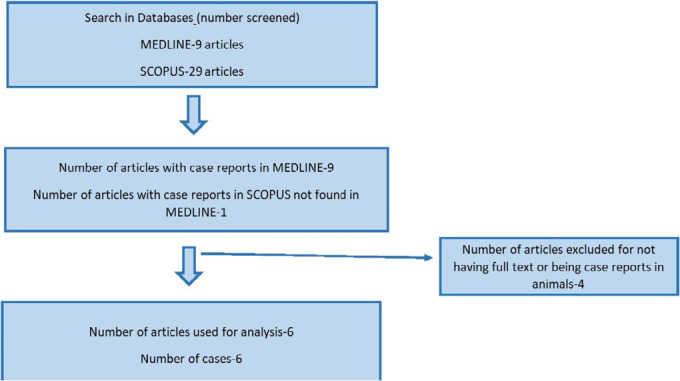
Search strategy and workflow chart.

Informed written consent was obtained from the patient. We followed the search strategy for writing review articles as proposed by Gasparyan et al.^3^ Inclusion criteria were cases of Histoplasma myositis published in MEDLINE or SCOPUS. Articles available on MEDLINE or SCOPUS published anytime were reviewed using search words {(“Histoplasma” [MeSH Terms] OR “Histoplasma” [All Fields]) AND (“myositis” [MeSH Terms] OR “myositis” [All Fields]). Thirty-eight articles were obtained. Articles in which full text was not available or which were not in English were excluded. On reviewing these articles, we found 6 with case reports (amounting to 6 cases).^4–9^ A flow chart of the search strategy is shown in **Figure 3**. The case published by Percy et al.^10^ and Salfelder et al.^11^ was excluded, as they described Histoplasmosis in animals. The studies published by Diongue K et al. and Yarzabal et al. were not included, because full text was not available. A search on disseminated Histoplasmosis in autopsy series was also conducted using MeSH terms Disseminated [All Fields] AND (“Histoplasmosis” [MeSH Terms] OR “Histoplasmosis” [All Fields]) AND (“Autopsy” [MeSH Terms] OR “Autopsy” [All Fields])). Gleaming through the 458 articles, 49 had Histoplasmosis in the title. Twenty-three articles were obtained wherein autopsy was done.

## DISCUSSION

Histoplasmosis is an endemic mycosis, most prevalent in North America, Ohio and Mississippi river valley, certain parts of central and south America. Histoplasma capsulatum, a thermally dimorphic fungus with mycelial and yeast forms is the primary pathogen.^12^ Mycelial forms with micro and macroconidia are the most common infectious form. Histoplasmosis usually occurs due to inhalation of fungal spores or reactivation of old infection. Disseminated Histoplasmosis often affects patients with defective cell-mediated immunity, post-transplant recipients, haematological malignancies, and those on immunosuppressive agents. Clinical manifestations can range from asymptomatic infection and acute pulmonary Histoplasmosis, to progressive disseminated Histoplasmosis depending on the intensity of exposure, immune status, and underlying lung architecture of host.^13^

Most common symptoms of disseminated Histoplasmosis include fever, hepatosplenomegaly, respiratory symptoms, lymphadenopathy, and weight loss. Muscle involvement is infrequent in histoplasmosis.^14^ After an extensive literature search, we could identify only 6 cases of Histoplasma myositis from 6 separate articles.^4–9^ A background of immunosuppression was present in all patients. Three out of the 6 patients were on glucocorticoids; 1 patient was on Etanercept therapy, and 1 patient had received a combination of Etanercept and glucocorticoid. The remaining 2 patients had HIV infection with low CD4 count. In addition to the muscle, fasciitis, cellulitis, and panniculitis were present in 3 and 1 each respectively. One patient had multifocal myositis. A background muscle disease that is inflammatory myositis was seen in 1 patient (**Table 2**, *Clinical Features*).

**Table 2.** Cases in Literature.

**Year**	**Author**	**Country**	**No of cases**	**Age/sex**	**Risk factors**	**Species**	**Underlying disease**	**clinical features**	**Treatment**	**Outcome**
1995	Voloshin et al. ^4^	USA	1	54 M	Corticosteroid Methotrexate	Histoplasma capsulatum	Dermato myositis	Myositis Necrotizing fasciitis	Iv amphotericin B Itraconazole	Improved
1996	Wagner et al. ^5^	USA	1	49 F	Azathioprine Methyl prednisolone	Histoplasma capsulatum	Chronic Renal failure, Post renal transplant	Necrotizing myofascitis	Iv amphotericin B Itraconazole	Death
2007	Goel D et al. ^6^	India	1	42 M	Low CD4	Histoplasma capsulatum	AIDS	Nodular myositis	Fluconazole	Improved
2009	Bourré-Tessier J et al. ^7^	Canada	1	67 F	Etanercept	Histoplasma capsulatum	RA	Focal myositis, panniculitis	Itraconazole	Improved
2015	Nimitvilai S et al. ^8^	Thailand	1	33 F	Low CD4	Histoplasma capsulatum	HIV	Myositis	IV amphotericin B itraconazole	Improved
2019	Silva et al. ^9^	Brazil	1	53 F	Etanercept corticosteroid	Histoplasma capsulatum	RA	Multifocal myositis Cellulitis, fasciitis	NA	NA

Majority of cases (66%) were associated with diffuse myalgia. Our patient had widespread myalgia at presentation, and new onset of diffuse myalgia in a patient of myositis on immunosuppressive therapy may be a pointer to suspect fungal infection of muscles. Such cases may warrant muscle imaging by ultrasound or MRI and muscle biopsy if imaging is inconclusive. As in the reported cases of Histoplasma myositis in literature, the patient described herein was on high dose glucocorticoids and developed steroid-induced diabetes mellitus. Such profound immunosuppression might have predisposed her to disseminated Histoplasmosis.

Out of the 5 cases in which treatment details were available, 3 cases received IV amphotericin B followed by maintenance therapy with oral Itraconazole. Two patients recovered, and 1 patient succumbed 5 months after the onset of illness. Our patient is into the eighth month of her illness, and currently on oral Itraconazole. However, persistent muscle weakness, in this case, could be attributed to anti-synthetase syndrome with inflammatory myositis which went undiagnosed until late. Anti-synthetase syndrome is a relatively uncommon entity with partial forms being more common, especially in anti-Jo1 + subset.^15^ The possibility of microangiopathy predisposing to opportunistic infections in dermatomyositis has been postulated previously in a case study by Voloshin et al.^4^

Similarly, pre-existing muscle inflammation might have predisposed this lady to Histoplasma pyomyositis.^17^ The exact mechanisms of immune-mediated injury in susceptibility to opportunistic infections and subsequent pyomyositis merit further research. Muscle involvement was absent in a systematic search of autopsy series in disseminated Histoplasmosis, suggesting the rarity of this case study.

Lastly, it is of prime importance to a have a high index of suspicion for fungal infiltration of muscles when a patient with inflammatory myositis on immunosuppression presents with worsening myalgias. The presence of ulcero-nodular skin lesions may serve as a clue. Anti-synthetase syndrome may be misdiagnosed as arthritis, liver disease or lupus. A cytoplasmic pattern of ANA in the presence of an appropriate clinical setting can help in making a diagnosis of the anti-synthetase syndrome.
